# P-2321. Role of the Transplant Infectious Disease Physician in Potential Donor Evaluation for Successful and Safe Abdominal Transplantation

**DOI:** 10.1093/ofid/ofae631.2473

**Published:** 2025-01-29

**Authors:** Nicholas DiStefano, Nikita Shah, Yoichiro Natori, Shweta Anjan, Giselle Guerra, Jacques Simkins, Julia Bini Viotti, Mahmoud Morsi, Eric Martin, Vighnesh Venkatatsamy

**Affiliations:** University of Miami Miller School of Medicine, Hollywood, Florida; University of Miami Miller School of Medicine, Hollywood, Florida; University of Miami, Miami Transplant Institute, Jackson Health System, Miami, Florida; University of Miami Miller School of Medicine and Miami Transplant Institute, Jackson Health System, Miami, Florida; Miami Transplant Institute, Jackson Health System, Miami, Florida; University of Miami Miller School of Medicine and Miami Transplant Institute, Jackson Health System, Miami, Florida; University of Miami/ Jackson Memorial Hospital, Miami, Florida; University of Miami, miami, Florida; Jackson Memorial Hospital/ Miami Transplant Institute, Miami, Florida; Miami transplant Institute, Miami, Florida

## Abstract

**Background:**

Preventing donor derived infections is critical in solid organ transplant medicine. However, to expand the donor pool, utilizing organs from donors with known infections or at risk of disease transmission with proper prophylaxis and/or treatments is necessary. The purpose of this study is to determine the efficacy and impact of robust transplant Infectious disease (TxID) evaluation for high-risk donors in solid organ transplant centers.

**Methods:**

This is a retrospective study conducted at one of the largest solid organ transplant centers in the US. All electrical medical records regarding donor information and obtained interventions were retrospectively reviewed between June 2023, and March 2024.

**Results:**

During study period, we performed 358 (250, 15, and 93 kidney, kidney-pancreas, and liver) transplants. At the time of organ offer, out of 4,579 total organ offers, 93(2.03%) TxID consultations occurred. The majority of TxID consults were to review positive culture (50/93, 53.8%) and abnormal imaging. TxID declined 19/93 (20.4%) cases due to high risk of infectious disease transmission such as active staphylococcal bacteremia or candidemia. Additional 44 cases were either declined due to other reasons or accepted in other centers. Finally, 30 cases were transplanted and out of those, 22 (73.3%) received prophylaxis. No donor derived infections were noted in those 30 cases. Of note, graft loss or death due to infection within 90 days post-transplant was seen in 10/250, 7/93, and 2/15 for kidney, kidney pancreas, and liver transplants respectively, and none of those cases developed donor derived disease.

**Conclusion:**

Solid organ transplantation helps prolong and improve recipients’ quality of life. Risk assessment, prophylaxis, and monitoring makes it possible to safely accept donors who are considered high risk for infection transmission, while preventing donor derived infections. Early TxID consultation for those cases is crucial.

**Disclosures:**

Yoichiro Natori, MD, Eurofin/Viracor: Honoraria|Nobel Pharma: Advisor/Consultant

